# Thin film of Poly(acrylic acid-*co*-allyl acrylate) as a Sacrificial Protective Layer for Hydrophilic Self Cleaning Glass

**DOI:** 10.3390/ma3053369

**Published:** 2010-05-25

**Authors:** Jānis Lejnieks, Ahmed Mourran, Walter Tillmann, Helmut Keul, Martin Möller

**Affiliations:** DWI an der RWTH Aachen e.V. and Institute of Technical and Macromolecular Chemistry, RWTH Aachen, Pauwelsstr. 8, D-52056 Aachen, Germany; E-Mails: lejnieks@dwi.rwth-aachen.de (J.L.); tillmann@dwi.rwth-aachen.de (W.T.)

**Keywords:** thin films, self-cleaning glass, ATRP, cross-linking reaction, hydrogels

## Abstract

Poly(acrylic acid-*co*-allyl acrylate) statistical copolymers were synthesized in a controlled manner in two steps: first *tert.*butyl acrylate and allyl acrylate were polymerized via atom transfer radical polymerization (ATRP) and afterwords the *tert*.butyl protective groups were removed via hydrolysis. Samples of self cleaning glass (SCG) were coated with thin films of poly(acrylic acid-*co*-allyl acrylate) and cross-linked afterwards by UV irradiation (in the presence of a photoinitiator and an accelerator). Solution cast thin films were transparent and homogeneous before and after UV cross-linking. The irradiated samples were found to be hydrophilic (Θ < 20°) and water insoluble. The coating prevented the spontaneous hydrophobization of the SCG by residual silicon exhaled from the sealing material. The TiO_2_ photocatalyst that covers the glass surface was found to strip the protective coating. The rate of the photooxidation process was measured by IR spectroscopy. The real field performance of the protective coating was also tested.

## 1. Introduction 

Progress in the understanding and fabrication of surfaces with controlled wetting properties allowed the emergence of contamination free surfaces (or “no clean”). A familiar example is the so-called Lotus effect or ultra-hydrophobic surface, which combines multi-scale surface roughness and non wetting surface chemistry [1]. On such surfaces, droplets have minuscule contact areas and drip off easily, taking powder-like contaminants along. Conversely, self cleaning mechanisms of ultra-hydrophilic surfaces rely on the flow of the liquid film. Ultra-hydrophilic surfaces are wetted easily: if the surface is inclined, it is the flowing liquid film that carries the dirt along [2,3,4,5]. 

Wettability, however, is not the only desired functionality: there are many additional requirements for surfaces in day-to-day use. For most glass applications, transparency or low scatter is essential. The ultra-hydrophobic approach to glass surfaces presents a fundamental problem: the substrate roughness may hinder transparency owing to scatter losses. For self cleaning glass surfaces based on the ultra-hydrophilic approach, the most interesting, and also with respect to applications the furthest developed route, has emerged by using TiO_2_ as a surface coating. TiO_2_ is an example of photocatalytically active metal oxide. Under exposure to ultraviolet light, it shows extremely small contact angles of less than 1° [6]. The origin of this light-caused wettability enhancement is not fully understood [7,8]. 

The main advantage of these surfaces is the combined hydrophilicity and photodegradation effect, which significantly aids in the cleaning process. Although the ultra-hydrophilic effect is reversible in principle, the ageing of these surfaces under real conditions is not known. Recently, effort has been taken to quantify the influence of the atmospheric pollutants e.g. soiling, and ageing on the self cleaning glass functionalities [9]. In particular, pollutants that leach from siloxane based resin, which is often used to seal the glass on a frame. Such contaminants are hydrophobic and promptly spread on ultra-hydrophilic surface, resulting in a thin hydrophobic layer, which may be detrimental to the TiO_2_ photocatalyst [10,11,12,13]. 

Leaching is a well known phenomenon in silicone based resins [8]. The reason is the occurrence of chemical degradation of the sealant in the first time after curing and release of siloxane oligomers during hardening that is specific for silicone chemistry. In general, formulations of curable silicone resins contain low molecular weight cross-linking agents, catalysts and plasticizers; a balance between cross-linking density and mechanical properties is often a challenge in formulation [14]. Besides that, additional processes like chain scission owing to the lability of the silicone carbon bond (Si—C) towards acidic and alkaline conditions may also occur during the initial exposure to environmental conditions. Moreover, spreading of the residual siloxane is favored on the hydrophilic SCG surface, since the TiO_2_ surface is a high-energy one and therefore should be wetted by most liquids in particular by the low surface tension siloxane [10]. Such contaminants not only are hydrophobizing the surface but are also known to hydrolyze and condense under the influence of ambient humidity and radiation to form thin layers of SiO_2_ [15]. It is this layer which irreversibly alters the photocatalytic activity of the TiO_2_.

In this work, we considered the possibility to prevent these hydrophobic contaminants by protecting the SCG surface with a thin sacrificial hydrogel layer. This requires a hydrophilic and water insoluble polymer layer with enhanced adhesion toward TiO_2_ support. The polarity of the hydrogel layer facilitates spreading of the siloxane residues and drastically reduces their adherence to the surface. However, since the TiO_2_ photocatalyst is actively decomposing the hydrogel layer, care has to be taken to match the photodecomposition rate of the protective layer to the leaching rate of the sealant. While adhesion and hydrophilicity can be easily fulfilled, controlled photodecomposition of the protective layer with respect to curing and ageing of the silicones resin is a challenging problem.

In this respect, we report on the design and synthesis of a new polymer - poly(acrylic acid-*co*-allyl acrylate) - that can be used to formulate a protective coating for SCG. The acrylic acid monomer has been selected because of its polarity and its spontaneous and specific binding to TiO_2_ through hydrogen bridges. It is demonstrated that incorporation of a UV cross-linkable unit along the polymeric chain will ensure water insolubility of the coating. Furthermore, the hydrophilicity and the performance of such a layer in protecting the SCG were tested in ambient light and in real atmospheric conditions.

## 2. Results and Discussion

Free radical polymerization and copolymerization of allyl acrylate (AA) with alkyl (meth)acrylates and styrene leads to a cross-linked polymer even at low conversion. This result is the consequence of the structure of AA with two reactive double bonds; a highly reactive C,C double bond in the acrylate - and a less reactive C,C double bond in the allyl group. In contrast, polymerization of AA and copolymerization of AA with alkyl acrylates using ATRP [16] offers the possibility to obtain soluble copolymers suitable for cross-linking in a later stage. The control over the polymerization does prevent the formation of an extended cross-linked network by the creation of more uniform chains [17].

Copolymerization of *tert*.butyl acrylate and allyl acrylate via ATRP was chosen because the method allows synthesizing polymers containing *tert*.butyl and allylic side groups in the same molecule. By removing the *tert*.butyl group, a highly hydrophilic copolymer - poly(acrylic acid-*co*-allyl acrylate) - is obtained with the potential of cross-linking due to the C,C double bonds in the allyl group. This copolymer cannot be obtained by direct copolymerization of acrylic acid with allyl acrylate because the acid monomers poison the ATRP catalyst by coordination of the transition metal or by protonation of the nitrogen atoms of the ligand [18]. 

**Scheme 1:**
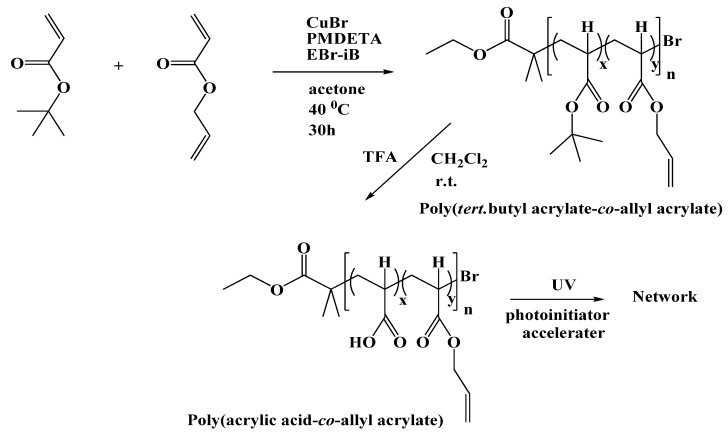
A Synthesis of poly(acrylic acid-*co*-allylacrylate), the linear precursor for the cross-linked hydrophilic polymer film.

In the first step the commercially available acrylic monomers were copolymerized in a controlled manner using ATRP (Scheme 1). The results are summarized in Table 2 (cf. experimental part).

In the first step, two linear precursors of poly(*tert*-butyl acrylate-*co*-allyl acrylate) with different ratio of the repeating units were successfully prepared: *t*BuA/AA: (95/5) and (90/10). The reproducibility of the reaction was proven by three-fold repetition of the experiment. 

^1^H-NMR analysis shows all expected signals: for the allylic groups at *δ* = 4.55 (–C**H_2_**–CH=CH_2_–), 5.23 (–CH_2_–CH=C**H_2_**) and 5.91 ppm (–CH_2_–C**H**=CH_2_–) and for the *tert*-butoxy- group (–OC(C**H**_3_)_3 _) at *δ* = 1.27 ppm. GPC analysis indicates that the copolymer has an average molecular weight of *circa* 5000 g/mol which is in very good agreement with the theoretical value. In addition a low polydispersity index (PDI = 1.23) was observed as expected for a controlled radical polymerization. Hydrolysis of the *tert.*butyl ester groups was performed in CH_2_Cl_2_ in presence of trifluoracetic acid (TFA) at room temperature for 24 h. ^1^H-NMR analysis clearly indicates that the signal derived from the *tert*-butoxy groups at *δ* = 1.27 ppm vanished and therefore the hydrolysis is quantitative. Moreover, during hydrolysis the copolymer becomes insoluble and precipitates spontaneously, allowing an easy isolation of the product as a white powder. 

### 2.1. Bulk reticulation

The efficacy of low amounts of allylic groups along the backbone to generate crosslinked material was tested. A defined amount of polymer poly(acrylic acid-*co*-allyl acrylate) 90/10 was dissolved in THF to yield a concentration of 0.1g/mL followed by addition of photo-initiator 2 wt % as well as the addition of an accelerator 3 wt %. Complete dissolution preventing cross-linking was ensured by stirring the mixture at room temperature in the dark. After evaporation of the solvent, the sample was irradiated for four hours with a 300 W UV lamp; the distance between sample and lamp was 30 cm. After that, THF was added to the solid material and kept overnight in the dark. The solvent was removed and the sample was dried at 50 °C for four hours. By weighing the solid material it was found that 82 wt % of the polymer was cross-linked. For thin film preparation, the polymer was cross-linked according to the same procedure. 

### 2.2. Cross-linked thin films

**Figure 1:**
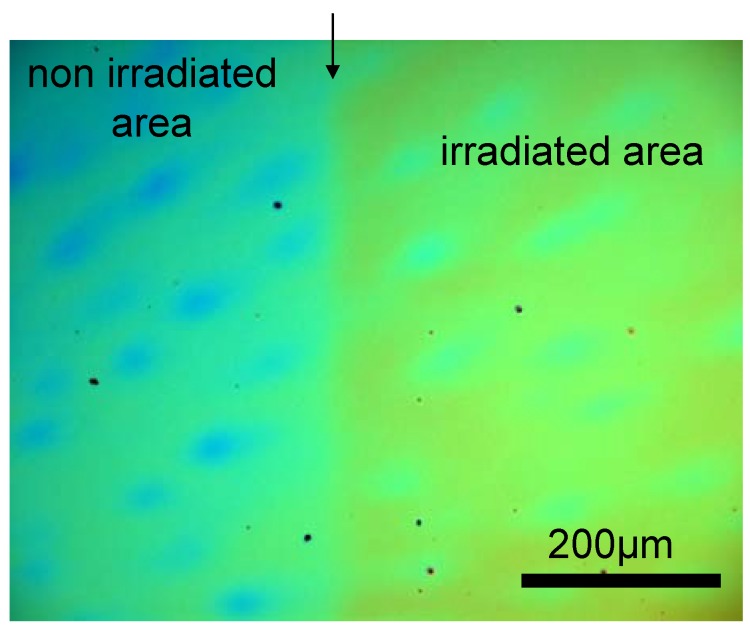
Optical micrograph of the surface of a thin poly(acrylic acid-*co*-allylacrylate) layer. (molar ratio 90/10). The film was cast from 10 wt % THF solution and irradiated with a UV lamp (6 mW/cm^2^) for 4 hours through a shadow mask. The arrow indicates the border between the irradiated and non-irradiated area.

First the film forming properties of poly(acrylic acid-*co*-allylacrylate)s was investigated. Coatings of the polymers onto SiO_2_ substrates were prepared by spin coating of solutions with increasing polymer concentration. Optical microscopy showed that the coating on a SiO_2_ substrate was smooth and homogenous before and after UV irradiation (Figure 1).

Essentially, coatings prepared from poly(acrylic acid-*co*-allyacrylate) (molar ratio 90/10) and long irradiation time yielded water insoluble coatings. Note that these cross-linking conditions are comparable to those of the bulk material.

Secondly, the influence of the copolymer composition, ratio of acrylic acid to allyl acrylate repeating units, on thin film reticulation was investigated. It is expected that the solid/air interface may reduce the cross-linking density near the interfaces, resulting in poor cross-linking efficiency and consequently poor film stability. To this end, we focused on qualitative analysis of the stability of thin films upon water droplet deposition. The variables are the molar content of the reactive groups in the copolymer, the photoinitiator and the accelerator concentration. Thin films of different molar compositions on SiO_2_ substrates were prepared by spin coating. The results are summarized in Table 1, in all cases homogeneous films were obtained before and after irradiation (see Figure 1). From Table 1, one can conclude that the optimum results in terms of the shortest irradiation time and the film insolubility are obtained with the urethane dimethacrylate as accelerator and a copolymer with a molar composition of acrylic acid to allyl acrylate of 90/10, respectively.

The hydrophilicity of the coating on a model substrate was tested by the sessile droplet method. Only contact angle measurements on water insoluble coatings are reported. The UV cross-linked coating exhibited a contact angle of less than 20°, suggesting a hydrophilic surface as expected for a thin hydrogel layer of polyacrylic acid. These results are in contrast to the literature data [19,20] on adsorbed or plasma grafted poly(acrylic acid) layers, which show a contact angle of 45°-50° and suggest that the cross-linked poly(acrylic acid) coating behaves like thin hydrogel layers.

**Table 1:** Water solubility of the coating: Effect of the ratio of repeating units, the photoinitiator, the accelerator and the irradiation time.

**Copolymer acrylic acid/allyl acrylat**	Photoinitiator ^a ^in wt %	Accelerator in wt %	Irradiation time in h	Optical Microscopy observation
**P(acrylic acid-*co*-AA) 95/5**	2	2 ^b^	2	soluble
	3	3 ^b^	4	partial soluble
**P(acrylic acid-*co*-AA) 90/10**	3	2 ^b^	2	partial soluble
	2	3 ^b^	4	insoluble
	2	5 ^c^	2	insoluble

^a^ 2,2 dimetoxy-2-phenylacetophenone; ^b^ Di-(ethylenglycol)-dimethacrylate; ^c^ urethane dimethacrylate: 7,7,9-trimethyl-4,13-dioxo-3,14-dioxa-5,12-diazahexadecane-1,16-diyl bis(2-methylacrylate).

A protective coating should preserve the optical transparency of the SCG. At this stage, we believed that coating with a thickness of *circa* 100 nm or less has minimum impact on the optical properties; the reason is the high refractive index of titanium dioxide layer (2.1–2.7) compared to a thin polymer coatings (1.45) [21]. The film thickness is readily variable through the polymer concentration and the casting processes, e.g., spin coating, dip coating or spraying. Coating processes are not the scope of this work, however, since we were interested in coating large glass surfaces efforts have been taken to show the feasibility of solution spraying as the most suitable technique. Therefore, we qualitatively assessed the influence of the polymer concentration as well as the deposition process mainly spin- coating *versus* spraying on the layer thickness.

**Figure 2:**
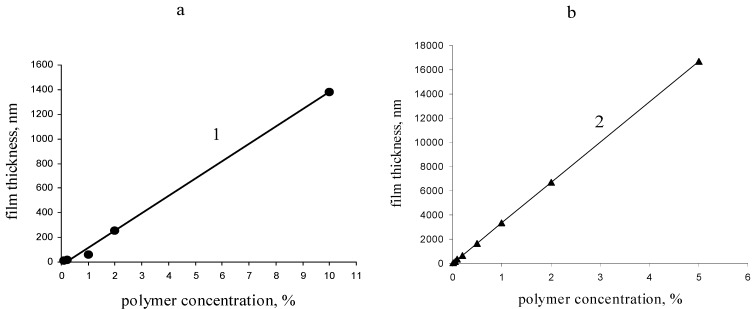
Film thickness as a function of polymer concentration used and processing conditions applied.** (a)** Film prepared by spin coating; film thickness measured by ellipsometry (1). **(b)** Film prepared by spraying and thickness estimated from the sprayed volume assuming that the polymer density is 1 g/cm^3^ (2).

Both processes showed a monotonic increase of the thickness with the polymer concentration. As expected, the difference between the two methods is the deposited amounts, which were higher for solution spraying (Figure 2). This is particularly true since the thickness was evaluated from the sprayed volume and the deposited amount varies with the pressure employed during the spraying process. The dependence illustrated in the right graph of Figure 2 should be regarded as tendency since we systematically overestimate the thickness. In contrast, the ellipsometry data on the spin coated films are more accurate. For instance increasing the concentration from 0.1 wt % to 5 wt % resulted in films with thickness of 10 nm and 500 nm, respectively.

The spin coating process is certainly not appropriate to coat large surfaces, however, the accuracy of the results demonstrate that the copolymer spreads evenly on the silicone oxide/air interface leading to a thin homogenous layer down to the nanometer scale. We anticipate that the copolymer behaves in a similar way on SCG since adsorption is known to take place by hydrogen bonding on the titanium dioxide providing a homogenous coating with good adhesion to the support [22,23].

### 2.3. Functionalities of the thin hydrogel layer

**Figure 3:**
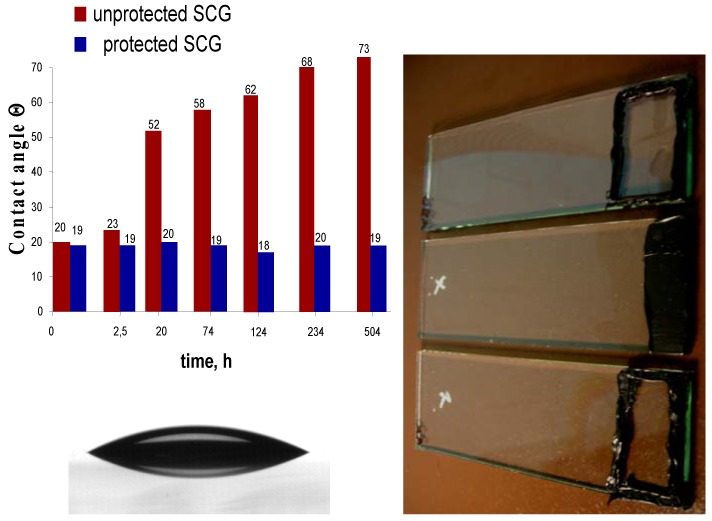
Effect of the sealant on the contact angle of a water droplet measured on protected and unprotected SCG as a function on time. (The origin denoted the time at which the silicone seal was applied.) On the right are photographs of the SCG samples wherein the sealants geometry (black) was varied. Below a side view of a water droplet on protected SCG is shown.

The polarity and the homogeneity of cross-linked copolymer coatings motivated us to further probe the ability of such layers to maintain the hydrophilic properties of SCG in presence of hydrophobic siloxane residues. Therefore solution of 2 wt % solid content were formulated and sprayed on activated SCG. To ensure that a water insoluble cross-linked polymer layer is formed the coated SCG was exposed for 2 hours to UV irradiation. Such treatment yielded in an optically homogenous hydrophilic surface with a water contact angle less than 20°. Subsequently two types of sealants were applied on the edge of the coated surface and the contact angle of a water droplet was measured as function of time. The resulting contact angles are plotted *versus* time (Figure 3; the origin denotes the time at which the silicon resin was applied). While the uncoated SCG becomes hydrophobic within a few hours after deposition of the sealant, the coated SCG preserves the initial hydrophilicity of the surface. Ageing of the sealant under ambient light and room temperature does not influence the results; the low contact angle of the coating on SCG persists over 20 days, whereas, the water contact angle of the uncoated SCG was constantly increasing with time which is consistent with our previous observations. Predominantly near the sealant within the first hour, the hydrophobic residues spread very rapidly to reach up to 10 cm after 20 hours. The performance of the protective copolymer layer in preventing the contamination of SCG surface by the hydrophobic siloxane residues has been demonstrated to last over a month with contact angle values that do not exceed 20°. It should be noted that this duration is longer compared to the curing time of the silicon resin.

To understand this result we considered the spreading parameter of siloxane residues on the two types of interface: Titanium dioxide/air and copolymer/air. 

It is well know that when a liquid drop is deposited on a solid, three interfaces come into play, and the three corresponding interfacial tensions: the solid-air interfacial tensions (γ_SA_), the solid-liquid interfacial tensions (γ_SL_), and the liquid-air interfacial tensions (γ_LA_), respectively. These interfacial tensions measure the free energy (per unit area) associated with an increase in the corresponding interface [24]. The spreading parameter ***S*** = γ_SA_- γ_SL_- γ_LA_ is the energy gained when covering one unit area of the dry solid with a flat liquid film of macroscopic thickness.

If ***S*** is negative, the situation where the solid is covered by a liquid film is not favorable. The equilibrium shape of the drop is a spherical cap, characterized by its equilibrium contact angle *θ,* the solution of Young’s equation cos*θ* = (γ_SA_- γ_SL_)/ γ_LA _[25].

If S is positive, the liquid spreads and tends to cover the maximum solid surface. Thus, S = 0 appears as criterion to distinguish between partial and total wetting [26]. 

Yet, if we consider the surface energy of the titanium dioxide as γ_TiO2/air_ ~ 280–380 mN/m [27,28,29] and assuming that the surface tension of the siloxane residue to be similar to polydimethylsiloxane PDMS γ_Siloxane/air _~ 18–20 mN/m, we conclude that the spreading parameter must be positive since the interfacial energy γ_TiO2/Siloxane_ cannot be larger than few tens of mN/m [29]. This is in agreement with the observed spontaneous contamination of the SCG surface by the siloxane residues (γ_TiO2Air _> γ_ TiO2/Siloxane_+ γ_Siloxane/air_). Following the same reasoning but replacing the titanium dioxide interface by the protective copolymer layer we get to the same conclusion (S > 0). Given that the surface energy of the copolymer protective layer is larger than that of the siloxane residue γ_PAA/air _~ 50–68 mN/m [30,31]; and the interfacial energy between the siloxane residues and the polyacrylic acid is below γ_PAA/Siloxane _~0.1 mN/m [32]. Thus spreading of the hydrophobic residues is expected to occur on the protective coating. 

**Figure 4:**
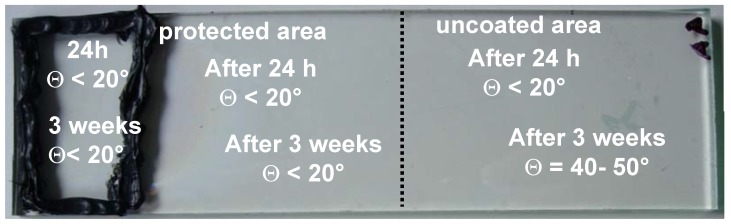
Partially protected SCG contaminated at the end of the protected area (black sealant): contact angles at different times from contamination. The discontinuous line indicates the end of the protective coating.

This was confirmed by coating half of the available glass surface with a protective layer; the layer was prepared as described before. The sealant was deposited only on the protected area in a way that the residual siloxane has to spread over the copolymer layer before contaminating the uncovered part of the surface. This is illustrated in Figure 4, wherein the resulting values of the water contact angle are indicated as a function of time. While the protected area was maintained hydrophilic, the bare SCG surface became hydrophobic after longer time as evidenced by the contact angle values. The effect was assigned to the siloxane residues that spread over the protective layer. These results comply with our understanding of the interfacial interactions, which favors the spreading of the low surface energy hydrophobic residues on both protected and unprotected hydrophilic SCG. More important is the fact that the siloxane residues are more weakly bonded to the copolymer layer (physisorbed) [33,34] than on the titanium dioxide surface [35,36]. Formation of a water film provokes desorption of the siloxane from the copolymer interface and favors their spreading on water air surface. This is likely what happened whenever we measured water contact angle on contaminated protective layer. As a consequence, the role of the copolymer layer is two-fold: it enhances the spreading of the hydrophobic residues and reduces their adhesion to the support. Both effects markedly contribute to facilitate removable of the silicone residues.

The viability of the approach was also supported by measuring the photodecomposition rate as a function of the radiation time for given film thickness. The rate of photooxidation of the protective layer was estimated by measuring the rate of decrease in the integrated IR absorbance associated with C-H stretching vibration under artificial sun light [37] (6 mW/cm^2^). 

**Figure 5:**
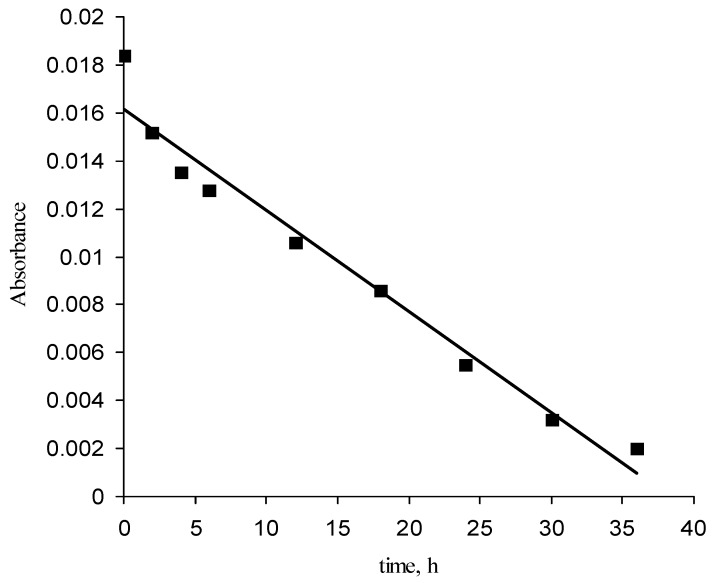
Intensity of the C-H vibration band as a function of UV irradiation time (the measurement was done on copolymer protective layer with thickness of 100 nm).

The results are displayed in Figure 5. It is shown that UV irradiation of a layer of 100 nm lasts up to 40 h. As expected, the protective layer is indeed not permanent. Decomposition occurs quite fast during the first four hours of irradiation. After this period, the concentration decreases almost linearly with time. If the initial amount of polymer is set equal to 100%, the average rate of decomposition can be calculated to be about 2.5% per hour. The accelerated test, however, provides only a hint on how long the layer will last until it photo-decomposes by the underlying TiO_2_ layer. From the model studies, it was demonstrated that under ambient light the protective layer persists for longer time and preserves its hydrophilicity up to 700 h.

In fact, a real field test was also considered on a large glass surface fixed on the frame by a silicone seal. The results of the field exposition, conducted at St-Gobain, are consistent with the model experiments and show that the hydrophilicity of the glass surface is preserved for more than one month. By comparison of the time needed for the silicone resin to cure and harden to the photodecomposition time of the protective layer we can fairly assume that the two processes are occurring in similar time scale.

## 3. Experimental Section

### 3.1. Materials

Allyl acrylate (AA, 85%, Lancester), *tert*.butyl acrylate (*t*-BuA, 99%, Acros), were filtered through an Al_2_O_3_ column before use. Ethyl-2-bromoisobutyrate (EBriB, 98%, Lancester), copper(I) bromide (>98%, Fluka), N,N,N’,N’’,N’’-pentamethyl diethylene triamine (PMDETA, >98%, Merck), acetone (99%, Aldrich ), trifluoro acetic acid (99%, Aldrich), 2,2-dimethoxy-2-phenylacetophenone (99%, Aldrich) were used as received.

All ATRP reactions were carried out in nitrogen atmosphere. Nitrogen (Linde 5.0) was passed over molecular sieves (4 Å) to eliminate traces of water.

### 3.2. Synthesis

#### 3.2.1. Copolymerization of tert-butylacrylate and allyl acrylate via ATRP

In a dry Schlenk tube acetone (6 mL), CuBr (1.11 mmol, 0.16 g), PMDETA (1.33 mmol, 0.23 g), *tert*.butyl acrylate (*t*-BuA) (39.0 mmol, 5 g) allyl acrylate (AA) (4.33 mmol, 0.57 g) and ethyl-2-bromoisobutyrate (EBriB) (1.11 mmol, 0.22 g), were mixed and stirred under nitrogen. The reaction was started by immersion of the Schlenk tube with the heterogeneous reaction mixture into an oil bath at 40 °C. After 30 h, the Schlenk tube with the dark green heterogeneous reaction mixture was cooled by a stream of cold water and diluted with CH_2_Cl_2_. To oxidize the copper complex the mixture was stirred in air. The oxidized copper complex was removed by adsorption on aluminium oxide and subsequent filtration. The obtained colourless filtrate was precipitated in a mixture of H_2_O/CH_3_OH (50/50). The polymer was obtained as a white powder. To prevent cross-linking the flask was protected with aluminium foil against light.

**Figure 6:**
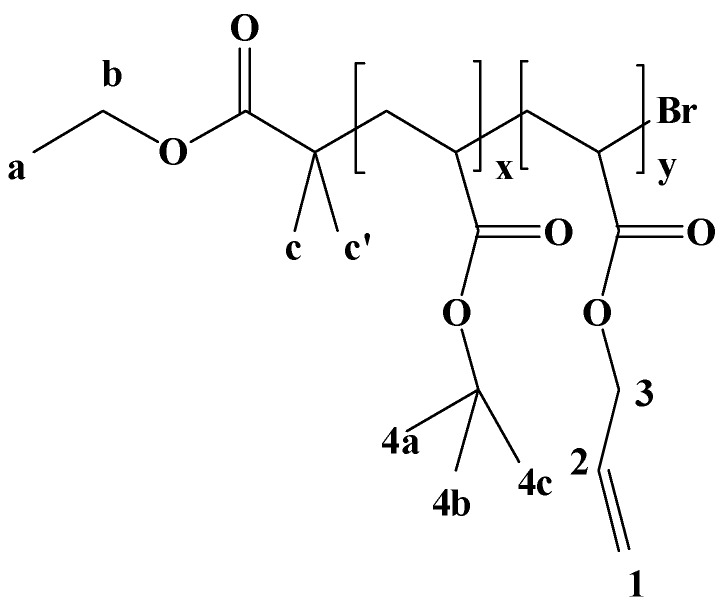
Structure of poly(*tert.*butyl acrylate-*co*-allyl acrylate) with ^1^H-NMR assignment.

^1^H-NMR (CDCl_3_): δ = 1.44 (broad s, 9H, *H-4a*, *H-4b*, *H-4c*); 4.55 (s, 2H, *H-3*); 5.20–5.34 (m, 2H, *H-1*); 5.88–5.92 (m, 1H, *H-2*) ppm.

The composition of poly(AA-*co*-*t*BuA) coresponds to the composition in the feed and is AA/*t*BuA = 10/90. Poly(AA-*co*-*t*BuA) with a ratio of AA/* t*BuA = 5/95 was obtained according to the same procedure using the composition in the feed given in Table 1. 

**Table 2:** Copolymerization of *tert*.butylacrylate (*t*.BuA) and allyl acrylate (AA): feed composition and product yield.

Polymer **AA/* t*-BuA**	Feed composition						Product		
	*t*-BuA	AA	CuBr	PMDETA	EBriB	acetone	m_exp_	Conversion %	M_n_
	mmol	mmol	mmol	mmol	mmol	mL	g	Yield %	PDI
P(AA-*co*-*t*BuA), **5/95**	39.0	2.10	1.10	1.30	1.1	6.0	1.6	46	5000
								30	1.23
P(AA-*co*-*t*BuA) **10/90**	39.0	4.33	1.11	1.33	1.11	5.0	2.0	49	4800
								36	1.33

#### 3.2.2. Removal of the tert.butyl protection group

Poly(*tert*.butyl acrylate-*co*-allyl acrylate) (1 g, 7.9 mmol repeating units) was dissolved in CH_2_Cl_2 _(20 mL). TFA (4.05 g, 35.5 mmol) was added slowly and the reaction mixture was stirred at room temperature for 24 h. The deprotected copolymer – poly(acrylic acid-*co*-allyl acrylate) precipitates from the reaction mixture. The product was filtered, then dissolved in THF and precipitated in hexane. After decantation and drying *invacuo* a white powder was obtained. Yield 82%.

**Figure 7:**
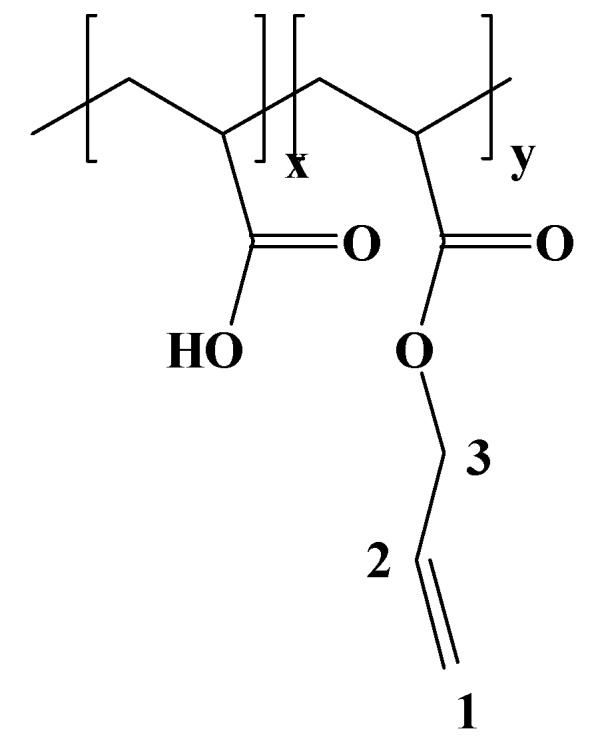
Structure of poly(acrylic acid-*co*-allyl acrylate) with ^1^H-NMR assignment.

^1^H-NMR (DMSO): δ = 4,49 (s, 2H, *H-3*); 5,17–5,28 (m, 2H, *H-1*); 5,79–5,84 (m, 1H, *H-2*) ppm.

#### 3.2.3. Measurements

NMR Spectroscopy: ^1^H and ^13^C NMR spectra were recorded on a Bruker DPX-300 FT-NMR spectrometer at 300 MHz and 75 MHz, respectively. Dimethyl sulfoxide (DMSO-*d_6_*) or chloroform (CDCl_3_) was used as the solvent. Tetramethyl silane (TMS) served as internal standard. 

Gel permeation chromatography: (GPC) analyses were carried out using a high pressure liquid chromatography pump (ERC HPLC 64200) and a refractive index detector (ERC 7515A) at 35 °C. The eluting solvent was tetrahydrofuran (THF) with 250 mg·L^-1^ 2,6-di-*tert*-butyl-4-methylphenol (Aldrich) and a flow rate of 1.0 mL·min^-1^. Four columns with MZ-DVB gel were applied. The length of each column was 300 mm, the diameter 8 mm, the diameter of the gel particles 5 μm, and the nominal pore widths were 50, 100, 1000 and 10,000 Å. Calibration with poly(methyl methacrylate) (PMMA) standards was used to estimate the molecular weights.

Film preparation: A defined amount of polymer was dissolved in THF and filtered through a 0.2 µm pore size filter prior use. The films were prepared either by spin coating or by spraying a dilute polymer solution onto substrates. To assess the layer homogeneity, the minimum thickness and the coating performance two types of substrates were studied. Silicone oxide as model surface since it has low roughness and high reflective index compared to SCG substrates. The thickness of the coating was controlled by the polymer concentration. Systematic variation of the polymer concentration from 10 wt % down to 0.1 wt % yields coatings that span the range from hundreds down to tens of nanometer.

Substrates and cleaning procedure: The self-cleaning glass SCG, activ and bioclean [38,39], was provided by Pilkington UK and Saint Gobain glass Germany, respectively. The Glass is coated with titanium dioxide (TiO_2_) photocatalyst in its nanocrystalline anatase form. TiO_2_ is deposited onto 4 mm float glass as a thin film (typically 10–20 nm) by an atmospheric pressure chemical vapor deposition technique, APCVD. A barrier layer of silicon oxide is necessary between the soda-lime silicate glass substrate and the deposited titanium dioxide layer to prevent alkaline metal ion migration between these two layers. The TiO_2 _free glass, Planilux, was provided by Saint Gobain Recherche (France). The glass sample has a rectangular shape with a size of (130 × 45) mm^2^. The glass was cleaned with detergent and extensively rinsed with demineralised water and methanol. The dried glass samples were exposed to UV irradiation for 4 hours. Systematically the contact angle of water droplet on the activated SCG surfaces was measured and found to be below 20°, which indicates the cleanness of the surface and the photo-activation of the TiO_2_ layer.

Silicone sealants: Silicone sealant is a curable resin designed for general weather-sealing. It is a silicone formulation that cures to a flexible rubber designed to seal the glass on a frame. Two types of sealants were studied, namely DC 791 and DC 756 provided by Dow Corning. According to the producer the two formulations differ in the working time: 15 min for DC791 and 30 min for DC 756, and in the time free tack: 35 min for DC 791 and 120 min for DC 756. Basically, this difference indicates to which extend the cross-linking reaction has been proceeded inducing increase of the viscosity and in the later curing time hardening. 

Deposition of curable silicone sealant on glass: Defined amount of sealant was applied at the edge of the SCG to cover a surface area of (10 × 45) mm^2^. The effect of the sealants on the SCG surface was assessed by measuring the variation of the water contact angle with ageing time at room temperature.

Optical microscopy: Observations were performed with an optical reflection microscope (Zeiss axioplan) equipped with a differential interference contrast (DIC) module. The micrograph was acquired with an Axio Cam digital camera.

Ellipsometry: Layer thicknesses were determined using a MM-SPEL-VIS ellipsometer from OMT. The silicon or Glass substrates were examined with a spectral method in the wavelength range from 450 to 900 nm. The azimuthal angle was kept at 15 degrees. The integration time was dependent on the layer thickness and the resulting signal intensity. The main source for systematic errors during the measurements is the correct position of the sample. This results in uncertainties in both the angle of incidence and the azimuthal angle. This error has been minimized by measuring all samples within one session right after another with exactly the same geometry of the device and positioning of the sample holder. Each single measurement averaged over the area of 3 × 5 mm. In addition, to further reduce systematic errors in the data collection, always one merely cleaned substrate was measured as reference in a series of experiments. Statistic errors are thus small but have, however been evaluated by measuring 5 different areas on each sample. The presented data are the average values of each sample and statistic errors for all samples were <1 nm

Sessile drop method: The contact angle measurements were carried out on a sessile drop G40 instrument (Krüss GmbH). With double distilled water as the wetting liquid, droplets were deposited onto substrates and the contact angle values were averaged over 10 droplets.

IR-Spectroscopy: Photodegradation kinetic of the protective layer was followed by FT-IR-spectroscopy. Spectra of the coated SCG samples were collected on a Nexus 470 spectrometer (Thermo Nicolet) using the transmission mode. As the spectral range below 2000 cm^-1^ is obscured by the very strong absorption of the glass only the C-H-stretching vibrations of the polymer around 2900 cm^-1^ could be observed. The band area was determined by drawing a baseline and integrating between 3040 and 2745 cm^-1^. For background subtraction we recorded spectra of the SCG without polymer coating. 

Photodegradation: The rate was estimated by measuring the rate of decrease in the integrated IR absorbance associated with the C-H stretching vibration of a solution cast film of poly(acrylic acid-*co*-allylacrylate) during sequential UV irradiation with light intensity of 5 mW/cm^2^ and wavelength range 290–800 nm. The photochemical process associated with UV irradiation was measured on coated conventional Planilux glass (TiO_2 _free) and found to be marginal with respect to the photocatalytic degradation. 

## 4. Conclusions

In conclusion, a concept of a sacrificial copolymer layer for the protection of hydrophilic self cleaning glass from silicone residues has been presented. The key step was to prepare a copolymer coating, which is UV cross-linkable, hydrophilic and water insoluble. We provided evidence that a random copolymer having the right molar composition of allyl acrylate and acrylic acid repeating units yield a thin hydrogel layer whose thickness is readily variable without alteration of the optical homogeneity neither the transparency of the coating. The adhesions of the copolymer on the TiO_2_ and cross-linking of the coating ensured stability of the layer against aqueous dissolution. Water droplets on such coatings adopt a low contact angle (below 20°). The hydrophilic surface of the protective layer favored spreading of the silicone residues and in the same time reduces their adhesion to the support. Under artificial sun light the protective layer undergoes photooxidation catalyzed by the TiO_2_. A thin sacrificial protective layer of 100 nm was stripped within 40 hours of UV irradiation. Such a coating is suitable for glassing application or as non-permanent hydrophilic coating.
